# Exercise Modulates Working Memory-Related Brain Activation Through Bidirectional Neural Mechanisms: An ALE Meta-Analysis of Longitudinal and Cross-Sectional fMRI Studies

**DOI:** 10.3390/brainsci16090963

**Published:** 2026-09-12

**Authors:** Jiayu Wang, Jun Wang, Wenshuang Tang

**Affiliations:** 1 School for Physical Education, Shanghai University of Sport, Shanghai 200438, China; wangjiayu@sus.edu.cn; 2 Faculty for Physical Education, Shanghai International Studies University, 550 Dalian West Road, Hongkou District, Shanghai 200083, China; 3 School of Psychology, Shanghai University of Sport, 399 Changhai Road, Yangpu District, Shanghai 200438, China

**Keywords:** exercise, working memory, functional magnetic resonance imaging (fMRI), activation likelihood estimation, cerebellum, task-positive network, default mode network

## Abstract

Background: Physical exercise improves working memory (WM) across the lifespan, yet the neural mechanisms underlying this benefit remain incompletely understood, and it is unclear whether intervention-induced neuroplastic changes and long-term exercise-related differences converge on common neural circuits. Methods: We systematically searched PubMed, Web of Science, PsycINFO, and CNKI through June 2026 and included 11 task-based fMRI studies (6 longitudinal interventions and 5 cross-sectional comparisons; 403 participants) reporting whole-brain activation coordinates. Activation likelihood estimation (ALE) meta-analyses were performed separately for activation increases and decreases within each design. Results: Longitudinal studies revealed exercise-induced activation increases in the bilateral cerebellum (posterior lobe, cerebellar tonsil) and decreases in the right thalamus. Cross-sectional studies revealed greater activation in the left middle temporal gyrus (BA 21) and reduced activation in the right cingulate gyrus (BA 24) and left caudate body among long-term exercisers relative to controls. Critically, the two designs yielded spatially non-overlapping patterns. Conclusions: These findings support a dual-mechanism model in which exercise strengthens task-positive network engagement while optimizing the suppression of task-irrelevant processing. They further suggest that exercise shapes working memory circuitry across distinct, timescale-dependent neural circuits.

## 1. Introduction

Working memory (WM)—the capacity to temporarily store and manipulate information—is fundamental to complex cognitive functions, including reasoning, problem-solving, and decision-making [1]. WM performance declines across the lifespan, with age-related deterioration particularly pronounced in older adults [2,3]. These declines significantly affect everyday functioning and quality of life, making the identification of effective intervention strategies a public health priority. The neural underpinnings of WM involve a distributed network encompassing prefrontal, parietal, and subcortical regions, which collectively support the maintenance and manipulation of information [4,5].

Mounting evidence suggests that physical exercise may mitigate WM decline across different age groups [6,7]. Aerobic training [6,8], resistance training [7,9], and mind-body exercises such as Tai Chi and yoga [10,11] have all been shown to improve WM performance in older adults, with similar benefits also observed in younger populations [12,13]. However, the neural mechanisms by which exercise exerts these cognitive benefits remain incompletely understood—and whether these effects manifest differently depending on the timescale of exercise engagement remains largely unexplored.

Studies investigating the neural basis of exercise-induced WM changes have employed two distinct designs, each capturing different temporal aspects of neuroplasticity. Longitudinal intervention studies assess changes in brain activation before and after a structured exercise program—ranging from acute single sessions to several months of training—capturing relatively short-term neuroplastic adaptations. In contrast, cross-sectional studies compare long-term exercisers or athletes with sedentary controls, reflecting stable neural characteristics associated with years of regular physical activity. While longitudinal designs offer causal evidence by tracking within-subject changes over time, they typically have shorter intervention periods and may not capture the cumulative effects of sustained exercise engagement. Cross-sectional designs can reveal differences associated with long-term exercise habits. However, they cannot establish causality and are susceptible to confounding factors such as genetics, lifestyle, or self-selection. Consequently, whether longitudinal and cross-sectional evidence converge on similar neural mechanisms or reflect distinct, timescale-dependent processes remains an open question.

Task-based functional magnetic resonance imaging (fMRI) studies have investigated exercise-induced changes in WM-related brain activation, but findings are inconsistent. Some studies report increased activation in prefrontal regions following exercise [14], while others report decreased activation in the cerebellum [15] or thalamus [16]. Furthermore, studies vary widely in design, participant populations, and WM tasks, making it difficult to synthesize findings. Activation Likelihood Estimation (ALE) meta-analysis provides a robust framework to quantitatively synthesize coordinate-based fMRI data and identify consistent activation patterns across studies [17], offering a principled approach to reconcile disparate findings.

To date, no ALE meta-analysis has systematically distinguished between longitudinal and cross-sectional evidence on exercise and WM-related brain activation. This distinction is critical: longitudinal studies capture exercise-induced neuroplastic changes, whereas cross-sectional studies reflect stable differences associated with long-term exercise habits. Moreover, exercise-related neuroplasticity may operate through distinct neural mechanisms that unfold over different timescales—from acute transient effects to chronic adaptations [18,19]. Therefore, the present study aimed to (1) identify consistent brain activation changes following exercise interventions in longitudinal studies; (2) identify consistent brain activation differences between long-term exercisers and controls in cross-sectional studies; and (3) compare the neural patterns revealed by the two study designs to evaluate whether longitudinal and cross-sectional evidence converge on similar neural circuits or reflect distinct mechanisms.

## 2. Materials and Methods

### 2.1. Protocol and Registration

This meta-analysis was conducted and reported in accordance with the Preferred Reporting Items for Systematic Reviews and Meta-Analyses (PRISMA) 2020 statement [20]. The completed PRISMA 2020 checklist is provided in Supplementary Materials. The study protocol was prospectively registered with the International Prospective Register of Systematic Reviews (PROSPERO; registration number CRD420261462524).

### 2.2. Literature Search

We systematically searched PubMed, Web of Science, PsycINFO, and the China National Knowledge Infrastructure (CNKI) for studies published through June 2026. The search strategy combined terms related to exercise, working memory, and fMRI. The complete search string was (‘exercise’ OR ‘physical activity’ OR ‘aerobic exercise’ OR ‘resistance training’) AND (‘working memory’ OR ‘N-back’ OR ‘n-back’) AND (‘fMRI’ OR ‘functional magnetic resonance imaging’ OR ‘BOLD’). To ensure that no eligible studies were overlooked, we additionally hand-searched the reference lists of relevant reviews and meta-analyses.

### 2.3. Inclusion and Exclusion Criteria

Studies were included if they met the following criteria: (1) Participants: healthy individuals without neurological or psychiatric disorders, cognitive impairment, or any conditions affecting brain function; (2) Study Design: original empirical research (reviews, meta-analyses, conference abstracts, and commentaries were excluded); (3) Intervention: for longitudinal studies, any form of structured exercise intervention, including aerobic exercise, resistance training, and multimodal exercise programs; for cross-sectional studies, comparisons between long-term exercisers/athletes and sedentary/non-exercising controls; (4) Cognitive Task: working memory was assessed using task-based fMRI with standard paradigms such as the N-back task; and (5) Outcome Reporting: whole-brain peak activation coordinates were reported in MNI or Talairach space.

Studies were excluded if they met any of the following criteria: (1) participants with neurological or psychiatric disorders, mild cognitive impairment, or any medical conditions affecting cognitive function; (2) non-exercise interventions such as cognitive training, meditation, or pharmacological interventions; (3) non-fMRI neuroimaging modalities (e.g., PET, DTI, structural MRI) or studies reporting only resting-state functional connectivity, brain network analyses, or region-of-interest (ROI) results without whole-brain coordinates; and (4) failure to report working memory-related fMRI activation results or failure to report whole-brain peak coordinates in MNI or Talairach space.

### 2.4. Study Selection and Data Extraction

Two reviewers (J.Y.W. and W.S.T.) independently screened titles, abstracts, and full texts against the inclusion criteria. Disagreements were resolved through discussion or consultation with a third reviewer (J.W.).

For each included study, the following information was extracted using a standardized data extraction form: (1) study characteristics: first author, year of publication, country; (2) participant characteristics: sample size, age, sex, education level, health status; (3) exercise intervention: type, intensity, frequency, duration (for longitudinal studies), or group definition (for cross-sectional studies); (4) fMRI task: working memory task type (e.g., N-back, Sternberg, change detection), task contrast, and experimental paradigm; (5) activation coordinates: peak activation coordinates in MNI or Talairach space, cluster sizes, and statistical thresholds; and (6) direction of effect: activation increase or decrease (post vs. pre-intervention for longitudinal studies; exercisers/athletes vs. controls for cross-sectional studies).

When multiple contrasts were reported within the same study (e.g., 1-back vs. 0-back, 2-back vs. 0-back), we extracted coordinates from the highest working memory load contrast (e.g., 2-back > 0-back) to maintain consistency. When studies report both whole-brain analysis and ROI results, only whole-brain coordinates were extracted. For studies that reported coordinates only in figures without tabulated values, we contacted the corresponding authors to request the coordinate data; if no response was received within four weeks, the study was excluded from the meta-analysis.

The specific working memory tasks employed across the included studies were as follows: (1) N-back task [21] (used in 7 studies): Participants monitor a continuous stream of stimuli and indicate whether the current stimulus matches the one presented n trials earlier. Load conditions included 0-back, 1-back, and 2-back. The primary contrast of interest was the highest working memory load minus the lowest load (e.g., 2-back > 0-back). (2) In-scanner working memory task [22] (used in 1 study; Sandstrom et al., 2025 [23]): This had a blocked design consisting of maintenance, manipulation, and control conditions. The primary contrast was manipulation > maintenance, reflecting active manipulation of information in working memory. (3) Sternberg working memory task [24] (used in 1 study; Gothe et al., 2018 [25]): Participants encode a set of letters, then indicate whether a subsequent probe letter was present in the original set. The contrast of interest was encoding > baseline. (4) Change detection task [26] (used in 1 study; Yao et al., 2024 [27]): Participants view an array of rectangles, and after a delay, indicate whether the orientation of one rectangle has changed. The contrast of interest was working memory load > null trials. (5) Two-Match task [28] (used in 1 study; Skolasinska et al., 2023 [29]): Participants continuously track two sets of numbers presented against different colored backgrounds, updating and switching between memory sets. The primary contrast was updating > non-updating trials. All tasks required the temporary maintenance and/or manipulation of information, consistent with the definition of working memory.

### 2.5. ALE Meta-Analysis

An ALE meta-analysis was performed using GingerALE 3.0.2 (http://www.brainmap.org/, accessed on 1 July 2026) [17,30,31]. For each experiment, reported coordinates were modeled as 3D Gaussian probability distributions with a full-width at half-maximum (FWHM) empirically determined based on sample size [30]. ALE maps were generated by computing the union of these probability distributions across experiments. Statistical significance was determined by comparing observed ALE scores against a null distribution generated from 5000 permutations. Clusters were thresholded at a voxel-level *p* < 0.001 (uncorrected) with a minimum cluster extent of 200 mm^3^, consistent with previous ALE meta-analyses in this field [5].

Separate ALE analyses were conducted for (1) longitudinal studies—activation increases (post > pre) and decreases (post < pre) and (2) cross-sectional studies—activation increases (exercisers > controls) and decreases (exercisers < controls). For visualization, significant ALE clusters were overlaid onto a standard MNI brain template using MRIcroGL (NITRC: MRIcroGL: Tool/Resource Info) [32]. Color bars indicate ALE values for each cluster.

## 3. Results

### 3.1. Study Selection and Characteristics

**Figure 1:**
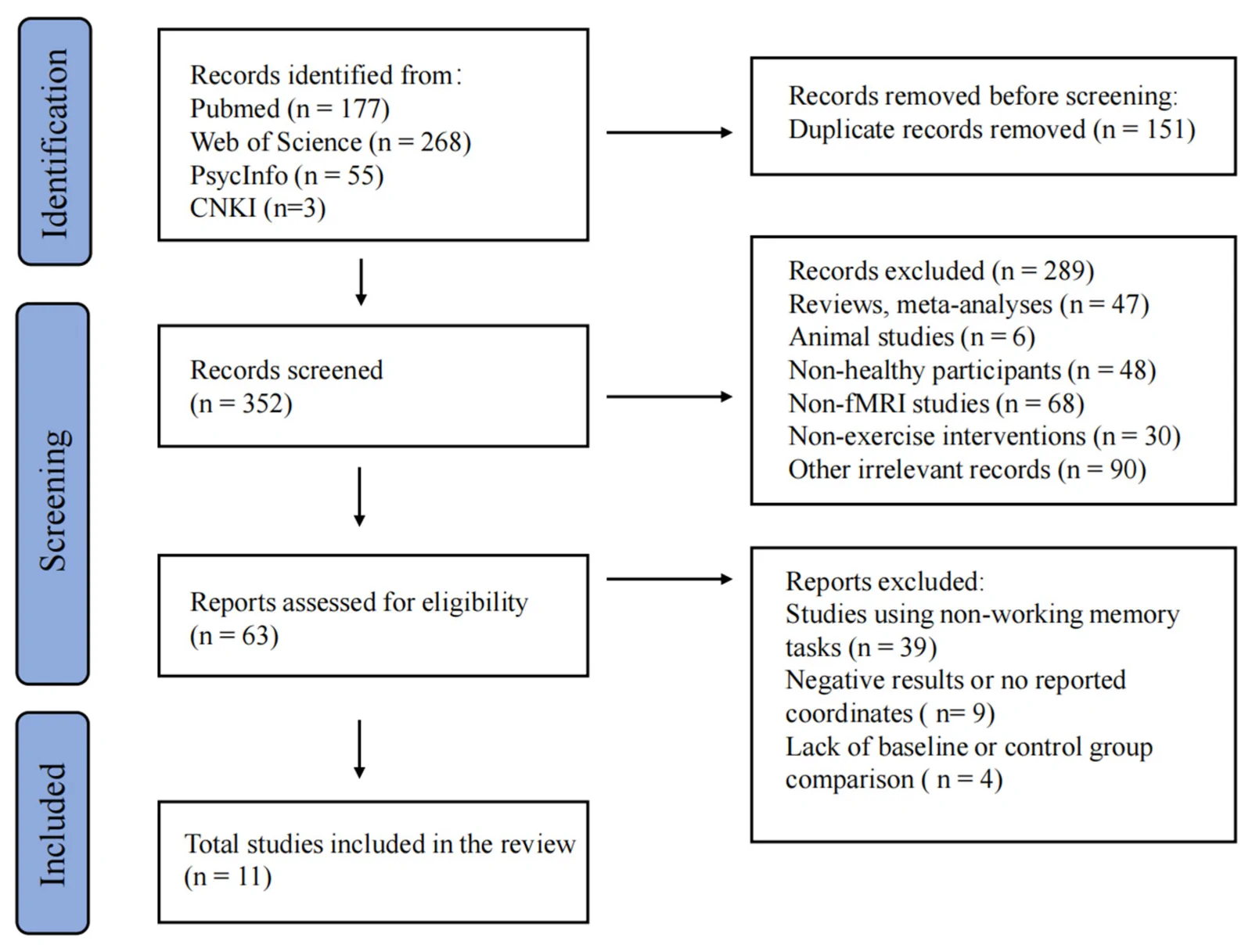
PRISMA 2020 flow diagram of the study selection process.

The systematic search of four electronic databases identified 503 records. After removal of 151 duplicates, title and abstract screening excluded 289 records that were clearly irrelevant. The main reasons for exclusion at this stage were reviews and meta-analyses (*n* = 47), animal studies (*n* = 6), non-healthy participants (*n* = 48), non-fMRI studies (*n* = 68), non-exercise interventions (*n* = 30), and other irrelevant records (*n* = 90). The remaining 63 full-text articles were assessed for eligibility, of which 52 were excluded for the following reasons: non-WM cognitive tasks (*n* = 39), resting-state fMRI, functional connectivity, or null results without reported activation coordinates (*n* = 9), and cross-sectional studies lacking an appropriate baseline or control group (*n* = 4). Ultimately, 11 studies met all inclusion criteria and were included in the ALE meta-analysis, comprising 6 longitudinal studies and 5 cross-sectional studies. The PRISMA flow diagram is presented in Figure 1. The completed PRISMA 2020 checklist is provided in Supplementary Materials.

The characteristics of the included studies are summarized in Table 1 and Table 2. Across the 11 included studies, a total of 403 participants (ranging from 9 to 110 per study) were included in the analyses, contributing 48 activation coordinates. A total of 158 participants were included in the longitudinal studies, with ages ranging from 10 to 74.7 years. Exercise interventions included 12-week Ai-Chi [15], 12-week multimodal exercise [16], 12-week high-intensity interval or moderate-intensity training [23], and acute cycling protocols [13,14,33]. Working memory tasks included the N-back task and an in-scanner working memory task. All studies reported coordinates in MNI space. Activation directions varied across studies, with some reporting increases and others decreases post-intervention.

For cross-sectional studies (Table 2), a total of 245 participants were included, with ages ranging from 20.8 to 70.6 years. Group comparisons included yoga experts versus sedentary controls [25], Olympic rowers/swimmers versus sedentary controls [27], basketball athletes versus non-athlete controls [34], high versus low strenuous physical activity [29], and physically active versus inactive individuals [35]. Working memory tasks included the Sternberg working memory task, change detection task, N-back task, and 2-Match task. All studies reported coordinates in MNI space, with activation directions varying across contrasts.

Notably, three studies reported bidirectional activation changes (i.e., both increases and decreases) across different brain regions. Specifically, Li et al. (2014) [14] reported both increased activation (right middle frontal gyrus, right lingual gyrus, and left fusiform gyrus) and decreased activation (anterior cingulate cortex, left inferior frontal gyrus, and right paracentral lobule) following acute exercise. Similarly, Yao et al. (2024) [27] observed that athletes showed increased activation in posterior parietal and temporal regions but decreased activation in frontal regions compared to controls. Qi et al. (2025) [34] also reported bidirectional patterns, with basketball athletes showing increased activation in task-positive network regions and decreased activation in default mode network regions relative to non-athlete controls. These bidirectional coordinates were extracted separately and classified according to their reported direction of effect for the subsequent ALE analyses.

### 3.2. ALE Meta-Analysis Results

From the 11 included articles, 48 coordinates were extracted, of which 27 came from longitudinal studies (14 coordinates that increased after exercise and 13 that decreased) and 21 came from cross-sectional studies (10 coordinates that increased and 11 that decreased in the exercise group compared to the control group). Separate ALE analyses were conducted for activation increases and decreases within each study design, yielding a total of 6 significant clusters. The detailed results are presented below.

#### 3.2.1. Longitudinal ALE Meta-Analysis Results

For the longitudinal studies examining exercise-induced changes in working memory-related brain activation, separate ALE analyses were conducted for activation increases and decreases.

**Figure 2:**
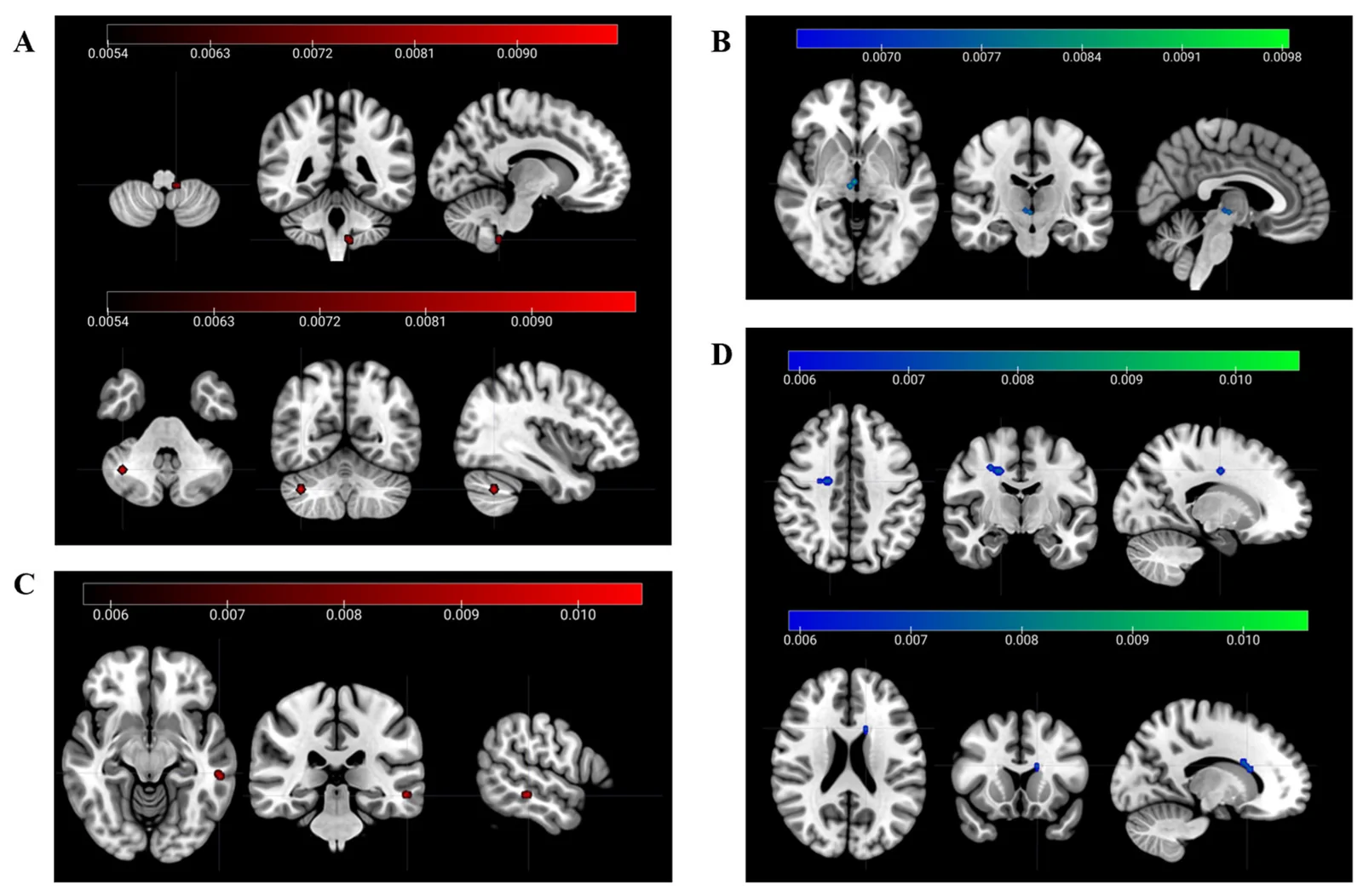
ALE meta-analysis results: (**A**) Longitudinal: activation increases (post > pre) in the bilateral cerebellum (posterior lobe, cerebellar tonsil). (**B**) Longitudinal: activation decreases (post < pre) in the right thalamus. (**C**) Cross-sectional: activation increases (exercisers > controls) in the left middle temporal gyrus (BA 21). (**D**) Cross-sectional: activation decreases (exercisers < controls) in the right cingulate gyrus (BA 24) and left caudate body. Clusters are overlaid on a standard MNI brain template. Color bars indicate ALE values. Red clusters indicate activation increases; blue clusters indicate activation decreases. Threshold: voxel-level *p* < 0.001 (uncorrected).

Activation increases (post > pre): The ALE analysis of exercise-induced activation increases revealed two significant clusters localized to the bilateral cerebellum (posterior lobe, cerebellar tonsil). The left cerebellar cluster was centered at MNI coordinates (−11, −44, −53) with an ALE value of 0.00878 (*p* = 2.77 × 10^−5^, Z = 4.03). The right cerebellar cluster was centered at (38, −60, −38) with an ALE value of 0.00988 (*p* = 2.86 × 10^−6^, Z = 4.54). Detailed results are presented in Table 3 and illustrated in Figure 2A.

Activation decreases (post < pre): The ALE analysis of exercise-induced activation decreases identified one significant cluster located in the right thalamus, with a peak at MNI coordinates (4, −18, −3). This cluster had an ALE value of 0.00738 (*p* = 6.11 × 10^−4^, Z = 3.23) with a volume of 304 mm^3^. Detailed results are presented in Table 3 and illustrated in Figure 2B.

#### 3.2.2. Cross-Sectional ALE Meta-Analysis Results

For cross-sectional studies comparing long-term exercisers with controls, separate ALE analyses were conducted for activation increases and decreases.

Activation increases (exercisers > controls): The ALE analysis of activation increases revealed one significant cluster in the left middle temporal gyrus (BA 21), with a peak at MNI coordinates (−57, −33, −10) and an ALE value of 0.01054 (*p* = 1.90 × 10^−5^, Z = 4.12). Detailed results are presented in Table 4 and illustrated in Figure 2C.

Activation decreases (exercisers < controls): The ALE analysis of activation decreases identified two significant clusters. The first cluster was located in the right cingulate gyrus (BA 24), with a peak at MNI coordinates (28, −8, 42) and an ALE value of 0.00803 (*p* = 6.96 × 10^−5^, Z = 3.81). The second cluster was located in the left caudate body, with a peak at MNI coordinates (−15, 15, 21) and an ALE value of 0.00762 (*p* = 3.15 × 10^−4^, Z = 3.42). Detailed results are presented in Table 4 and illustrated in Figure 2D.

### 3.3. Comparison Between Longitudinal and Cross-Sectional Findings

The brain regions identified in the longitudinal and cross-sectional analyses did not overlap spatially: longitudinal studies revealed changes in the cerebellum and thalamus, whereas cross-sectional studies revealed differences in the middle temporal gyrus, cingulate gyrus, and caudate body. This spatial dissociation suggests that exercise-induced neuroplastic changes (longitudinal) and long-term exercise-related differences (cross-sectional) may engage distinct neural circuits. A formal conjunction or contrast analysis between the two designs was not performed because the limited number of experiments per analysis would render such comparisons statistically unreliable [36] (see Section 4).

## 4. Discussion

This ALE meta-analysis quantitatively synthesized task-based fMRI evidence on how exercise relates to brain activation during WM tasks, separately examining intervention-induced changes (longitudinal studies) and long-term exercise-related differences (cross-sectional studies). Longitudinal studies revealed increased activation in the bilateral posterior cerebellum and decreased activation in the right thalamus following acute or short-term exercise, whereas cross-sectional studies revealed increased activation in the left middle temporal gyrus and decreased activation in the right cingulate gyrus and left caudate body in long-term exercisers relative to controls. Notably, the two designs yielded spatially non-overlapping patterns. Taken together, the present results suggest that exercise modulates WM-related brain networks through bidirectional mechanisms, strengthening task-relevant processing while optimizing the suppression of task-irrelevant processing. These changes may engage distinct neural circuits at different timescales of exercise engagement.

The longitudinal ALE analysis revealed exercise-induced activation increases in the bilateral posterior cerebellum, consistent with the growing recognition of the cerebellum’s contribution to higher-order cognitive functions, including WM [37,38]. The cerebellum is involved in WM through connections with frontal and parietal regions via the dentate gyrus–thalamus–cortex pathway [39]. The increased activation of the cerebellum following exercise may reflect an enhanced capacity to mobilize neural resources within the task-positive network (TPN)—a pattern consistent with the neural compensation hypothesis [40].

The TPN comprises frontoparietal and cingulo-opercular regions that support goal-directed cognitive processing, including attention, WM maintenance, and cognitive control [41,42]. Within the cerebellum, posterior lobules VI and VII (including Crus I) are consistently engaged during WM tasks requiring manipulation and cognitive control [43]. It should be noted, however, that cerebellar functional organization is not homogeneous: intrinsic connectivity mapping indicates that more inferior posterior regions, including lobule IX (the cerebellar tonsil), are preferentially coupled with the default mode network (DMN) rather than frontoparietal control systems [44]. The increased tonsil activation observed in our longitudinal analysis may therefore reflect not simply enhanced TPN engagement, but a recalibration of cerebellar participation in TPN–DMN interactions following exercise—for example, more effective cerebellar support of internally directed processing during task performance. This interpretation aligns with models of the cerebellum as a domain-general modulator that optimizes both task-relevant and task-irrelevant processing streams through a common predictive computation [39].

Similarly, the cross-sectional finding of increased left middle temporal gyrus (BA 21) activation in long-term exercisers compared to controls could suggest enhanced semantic and verbal processing strategies during working memory tasks. The middle temporal gyrus is involved in semantic processing and verbal working memory maintenance [45,46]. Its increased activation in athletes and physically active individuals indicates that long-term exercise may be associated with more efficient verbal encoding or strategy use during working memory tasks—a form of domain-specific cognitive enhancement that complements TPN engagement.

Complementing the activation increases, our analyses revealed decreased activation in the right thalamus (longitudinal) and in the right cingulate gyrus (BA 24) and left caudate body (cross-sectional). These regions participate in attentional gating, performance monitoring, and cognitive control, and their reduced activation can be interpreted within the framework of neural efficiency.

The DMN, comprising the medial prefrontal cortex, posterior cingulate cortex, angular gyrus, and middle temporal gyrus, is typically deactivated during externally oriented cognitive tasks [47,48]. Successful working memory performance depends on the ability to suppress DMN activity to minimize task-irrelevant internal mentation [49]. The decreased activation observed in the cingulate gyrus (BA 24), a core DMN node involved in conflict monitoring and attentional control [50], suggests that exercise may enhance DMN suppression—a key mechanism for improving cognitive efficiency.

The thalamus serves as a relay station in cortico-thalamo-cortical loops critical for working memory [51]. Reduced thalamic activation following exercise may indicate more efficient information transmission between cortical regions, requiring less thalamic gating to maintain task performance. Similarly, decreased activation in the caudate body—a key node of the cortico-basal ganglia-thalamic loop involved in working memory gating and updating [52,53]—indicates optimized cognitive gating mechanisms in long-term exercisers. These patterns collectively point to a pattern of neural efficiency: Fewer neural resources are required to achieve comparable cognitive performance following exercise training [54].

Taken together, our findings can be integrated within a dual-mechanism model of exercise-induced working memory improvement. Exercise appears to simultaneously enhance task-positive network engagement (increased cerebellar activation) and optimize DMN suppression (decreased thalamic, cingulate, and caudate activation). This TPN-DMN antagonistic relationship is well-established in cognitive neuroscience: during cognitively demanding tasks, TPN regions increase their activity while DMN regions decrease their activity [42]. The degree of this anticorrelation has been positively associated with better working memory performance and cognitive control [55,56]. The present findings extend this framework to the exercise domain. They demonstrate that both directions of activation change can be observed across different brain regions following exercise.

This dual pattern also resonates with prominent models of neurocognitive aging. The compensation hypothesis posits that older adults recruit additional neural resources to maintain performance in the face of age-related decline [40,57], whereas the scaffolding theory of aging and cognition (STAC) and its revised version (STAC-r) propose that the brain constructs supplementary circuitry in response to functional challenge, with exercise identified as a key enriching factor that promotes scaffolding [58]. The bidirectional activation changes observed here—enhanced recruitment of task-relevant regions alongside more efficient suppression of task-irrelevant activity—may represent the functional signature of such compensatory scaffolding driven by exercise.

A notable finding is the spatial dissociation between longitudinal and cross-sectional results. Longitudinal studies—capturing relatively short-term exercise effects ranging from a single acute session to several weeks or months of training—revealed changes in subcortical structures (cerebellum, thalamus). Cross-sectional studies—reflecting long-term exercise habits accumulated over years—revealed differences in cortical and striatal regions (middle temporal gyrus, cingulate, and caudate). This dissociation suggests that exercise impacts the brain at multiple levels and timescales.

One possible interpretation is that early exercise-induced neuroplastic changes predominantly affect subcortical nodes of the working memory network, while long-term adaptations extend to cortical regions. This pattern is consistent with the scaffolding theory of aging and cognition [59], which proposes that the brain engages additional neural circuits to maintain cognitive performance as demands and experiences accumulate over time. Alternatively, the dissociation may represent different underlying mechanisms: longitudinal studies capture state-like changes following training, whereas cross-sectional studies capture trait-like differences associated with sustained exercise habits.

It should be noted that the dual-mechanism model proposed here is based on a relatively small number of studies (6 longitudinal, 5 cross-sectional). While the spatial dissociation between longitudinal and cross-sectional findings supports the existence of distinct timescale-dependent mechanisms, future studies with larger sample sizes and more standardized designs are needed to directly test this model. Specifically, longitudinal studies with long-term follow-up and cross-sectional studies with well-matched control groups would help confirm whether exercise truly enhances TPN engagement while optimizing DMN suppression.

Several limitations should be acknowledged. First, the number of included studies was relatively small (6 longitudinal, 5 cross-sectional), reflecting the limited literature in this area. Second, the ALE analyses were thresholded at an uncorrected voxel-level *p* < 0.001, which, while consistent with previous ALE studies, may increase the risk of false positives. Third, the included studies varied in participant characteristics, exercise interventions, and WM tasks, introducing clinical heterogeneity. Fourth, due to insufficient data, we were unable to separate long-term exercise training from acute exercise effects in the longitudinal analysis. Fifth, due to the limited number of included studies and their substantial demographic heterogeneity, we were unable to perform subgroup analyses or meta-regression to examine the potential moderating effects of age, sex, or exercise type. Given that working memory and its neural correlates are known to vary with age and sex, future studies with larger sample sizes and more balanced demographic distributions are needed to determine whether the observed neural patterns are consistent across different demographic groups and exercise modalities.

## 5. Conclusions

This ALE meta-analysis provides quantitative evidence that exercise modulates WM-related brain activation through bidirectional neural mechanisms. Longitudinal studies revealed increased activation in the posterior cerebellum and decreased activation in the thalamus following acute or short-term exercise, whereas cross-sectional studies revealed increased activation in the middle temporal gyrus and decreased activation in the cingulate gyrus and caudate body in long-term exercisers. These patterns are consistent with a dual-mechanism model in which exercise enhances the engagement of task-relevant circuitry while optimizing the suppression of task-irrelevant processing, and the spatial dissociation between designs supports the interpretation that these mechanisms unfold across distinct, timescale-dependent neural circuits. Given the limited number of contributing experiments, these findings should be considered exploratory; they nonetheless provide a testable framework for understanding how exercise shapes the functional architecture of working memory across the lifespan.

## Figures and Tables

**Table 1:** Characteristics of longitudinal and acute pre–post studies included in the ALE meta-analysis.

Study	Sample Size	Mean Age (Years)	Female (%)	Exercise Intervention	Task	Foci	Space	Direction
Nissim et al., 2021 [15]	19	74.7	63.2	12-week Ai-Chi	N-back	3	MNI	↓
Nishiguchi et al., 2015 [16]	48	73.0	45.8	12-week multimodal exercise	N-back	7	MNI	↓
Sandström et al., 2025 [23]	43	69.8	55.8	12-week high-intensity interval or moderate-intensity training	In-scanner WM task	1	MNI	↑
Li et al., 2014 [14]	15	19.6	100.0	Acute cycling (20 min, moderate intensity)	N-back	6	MNI	↓ and ↑
Li et al., 2019 [33]	24	25.5	100.0	Acute cycling (30 min, moderate intensity)	N-back	5	MNI	↑
Chen et al., 2016 [13]	9	10	44.4	Acute cycling (30 min, moderate intensity)	N-back	5	MNI	↑

↑ = activation increase (post > pre); ↓ = activation decrease (post < pre); ↑ and ↓ = coordinates for both activation increases and decreases are available; Foci = number of coordinates extracted for ALE analysis; WM = working memory.

**Table 2:** Characteristics of cross-sectional studies included in the ALE meta-analysis.

Study	Sample Size	Mean Age (Years)	Female (%)	Group Comparison	Task & Contrast	Foci	Space	Direction
Gothe et al., 2018 [25]	26	35.8	92.3	Yoga experts vs. Sedentary controls	Sternberg WM task	1	MNI	↓
Yao et al., 2024 [27]	28	26.0	50	Olympic rowers/swimmers vs. Sedentary controls	Change detection WM task	6	MNI	↑ and ↓
Qi et al., 2025 [34]	110	20.8	44	Basketball athletes vs. non-athlete controls	N-back	5	MNI	↑ and ↓
Skolasinska et al., 2023 [29]	40	Older: 70.6 Younger: 26.3	60	High vs. low strenuous physical activity	2-Match task	3	MNI	↑
Song et al., 2025 [35]	41	Older: 63.3 Younger: 23.0	44	Physically active vs. inactive	N-back	6	MNI	↓

↑ = activation increase in athlete/high-fit group relative to controls/low-fit group; ↓ = activation decrease; ↑ and ↓ = coordinates for both activation increases and decreases are available; Foci = number of coordinates extracted for ALE analysis; WM = working memory.

**Table 3:** Longitudinal and acute pre–post studies ALE meta-analysis results: Exercise-induced changes in working memory-related brain activation.

Brain Region	Hemisphere	BA	MNI Coordinates			Volume (mm^3^)	ALE Value (×10^−2^)	*p* Value
			x	y	z			
activation increases								
Cerebellum (Posterior Lobe, Tonsil)	L	-	−11	−44	−53	336	0.878	2.77 × 10^−5^
Cerebellum (Posterior Lobe, Tonsil)	R	-	38	−60	−38	304	0.988	2.86 × 10^−6^
activation decreases								
Thalamus	R	-	4	−18	−3	304	0.738	6.11 × 10^−4^

Clusters thresholded at voxel-level *p* < 0.001 (uncorrected); BA = Brodmann area; L = left; R = right; MNI = Montreal Neurological Institute.

**Table 4:** Cross-sectional ALE meta-analysis results: long-term exercise-related differences in working memory-related brain activation.

Brain Region	Hemisphere	BA	MNI Coordinates			Volume (mm^3^)	ALE Value (×10^−2^)	*p* Value
			x	y	z			
activation increases								
Middle Temporal Gyrus	L	21	−57	−33	−10	336	1.05	1.90 × 10^−5^
activation decreases								
Cingulate Gyrus	R	24	28	−8	42	512	0.803	6.96 × 10^−5^
Caudate Body	L	-	−15	15	21	352	0.762	3.15 × 10^−4^

Clusters thresholded at voxel-level *p* < 0.001 (uncorrected); BA = Brodmann area; L = left; R = right; MNI = Montreal Neurological Institute.

## Data Availability

No new data were created in this study. All activation coordinates were extracted from the published articles listed in Table 1 and Table 2. The ALE input files and thresholded maps are available from the corresponding authors upon reasonable request.

## Supplementary Materials

The following supporting information can be downloaded at: https://www.mdpi.com/article/10.3390/brainsci16090963/s1, File S1: PRISMA 2020 Main Checklist.

## Abbreviations

The following abbreviations are used in this manuscript:

ALE	Activation Likelihood Estimation
DMN	Default Mode Network
fMRI	functional Magnetic Resonance Imaging
MNI	Montreal Neurological Institute
TPN	Task-Positive Network
WM	Working Memory
